# Optimization of the radiation dosimetry protocol in Lutetium-177-PSMA therapy: toward clinical implementation

**DOI:** 10.1186/s13550-023-00952-z

**Published:** 2023-01-24

**Authors:** Steffie M. B. Peters, Maaike C. T. Mink, Bastiaan M. Privé, Maarten de Bakker, Frank de Lange, Constantijn H. J. Muselaers, Niven Mehra, J. Alfred Witjes, Martin Gotthardt, James Nagarajah, Mark W. Konijnenberg

**Affiliations:** 1 Department of Medical Imaging, Radboud University Medical Center, P.O. Box 9101, 6500 HB Nijmegen, The Netherlands; 2 Department of Physics and Astronomy, Radboud University, Nijmegen, The Netherlands; 3 Department of Urology, Radboud University Medical Center, Nijmegen, The Netherlands; 4 Department of Medical Oncology, Radboud University Medical Center, Nijmegen, The Netherlands; 5 Department of Radiology and Nuclear Medicine, Erasmus Medical Center, Rotterdam, The Netherlands

**Keywords:** [^177^Lu]Lu-PSMA, Dosimetry, Radionuclide therapy, Prostate cancer, mHSPC

## Abstract

**Background:**

Dosimetry in [^177^Lu]Lu-PSMA therapy is a valuable tool to assess treatment efficacy and toxicity. This study aims to develop a clinically implementable protocol to determine the absorbed dose in organs and tumor lesions after [^177^Lu]Lu-PSMA-617 therapy, by reducing the imaging time points and utilizing population-based kinetics with a single scan, with evaluation of its influence on the uncertainty in absorbed dose.

**Methods:**

Ten patients with metastatic hormone-sensitive prostate cancer received two cycles of [^177^Lu]Lu-PSMA-617. Post-treatment imaging was performed at 1 h, 24 h, 48 h, 72 h and 168 h, consisting of three-bed positions SPECT/CT and a whole-body planar scan. Five-time point SPECT dosimetry was performed for lesions and organs with physiological uptake (kidneys, liver and salivary glands) and used as the reference standard. Absorbed dose values for various simplified protocols were compared to the reference standard.

**Results:**

Accurate lesion dosimetry is possible using one-time point SPECT imaging at 168 h, with an increase in uncertainty (20% vs. 14% for the reference standard). By including a second time point, uncertainty was comparable to the reference standard (13%). Organ dosimetry can be performed using a single SPECT at 24 h or 48 h. Dosimetry based on planar scans did not provide accurate dose estimations.

**Conclusion:**

Accurate lesion dosimetry in [^177^Lu]Lu-PSMA therapy can be performed using a one- or two-time point protocol, making dosimetry assessments more suitable for routine clinical implementation, although dosimetry based om multiple time points is more accurate.

*Clinical trial registration* This study was approved by the Medical Review Ethics Committee Region Arnhem-Nijmegen on January 23, 2018 and was registered on clinicaltrials.gov (NCT03828838).

**Supplementary Information:**

The online version contains supplementary material available at 10.1186/s13550-023-00952-z.

## Introduction

[^177^Lu]Lu-PSMA radioligand therapy is increasingly applied in metastasized prostate cancer patients [1–8]. A recent phase III study in patients with castration-resistant prostate cancer reported that both the progression-free survival and overall survival are significantly improved with [^177^Lu]Lu-PSMA-617 therapy [9]. The most common adverse events reported were fatigue, (mild) dry mouth and nausea. Moreover, the incidence of thrombocytopenia and lymphopenia was about three times higher in the treated group compared to the control group, indicating bone marrow toxicity is an important concern in these heavily pre-treated patients. Nevertheless, it is expected that upon registration of [^177^Lu]Lu-PSMA-617, the therapy will be implemented as standard of care in metastatic prostate cancer (mPC) with a large number of patients eligible for this treatment. However, despite selecting patients based on the level of PSMA uptake on PET scans, only about 50% of the patients showed a prostate-specific antigen (PSA) response (decrease of > 50%). This suggests that patient selection based on PSMA binding in target lesions alone is not sufficient. By calculating absorbed doses delivered to lesions, patient selection and treatment optimization might be improved. Moreover, pre- or intra-therapeutic dosimetry could help to reduce toxicity in organs at risk (e.g., kidneys and salivary glands [10–14]). This is especially valuable in patients with impaired function of the organs at risk, in patients that were previously treated with radioligand therapy, and in early-stage patients that have a longer life expectancy and thus might suffer from the late onset of organ toxicity.

While dosimetry is generally accepted as a valuable tool to assess tumor doses and organ toxicity, implementation into a clinical routine is difficult due to practical considerations such as patient burden (caused by repeated scanning), hospital resources and availability of suitable tracers. Therefore, most [^177^Lu]Lu-PSMA dosimetry studies performed either used planar scans instead of 3D SPECT [12, 13, 15, 16], acquired only a limited number of imaging time points [10] and/or focused on early time points only [10, 12]. A recent dosimetry study in metastatic hormone-sensitive prostate cancer (mHSPC) patients used five-time point SPECT/CT imaging and included a late time point at 7 days. This study was able to properly sample the radiotracer uptake time-activity-curve, however, it required significant hospital resources and was time-consuming for patients [14]. Hence, for dosimetry to become clinical routine, it is pivotal to simplify the imaging protocol without compromising the accuracy of the dose calculations.

Based on the data of the abovementioned study in HSPC patients, this study aimed to develop a routinely implementable protocol while adhering to the accuracy and uncertainty of the dose calculations. We hypothesized that the dosimetry protocol can be optimized by reducing the number of scanning time points and the number of scans per time point, as was also found for similar studies regarding [^177^Lu]Lu-DOTATATE studies for treatment of neuroendocrine tumors [17, 18]. Absorbed doses from simplified protocols were compared to the absorbed dose based on the elaborate reference imaging protocol (five-time point SPECT) to determine whether the simplification could provide a reliable alternative, taking into account both accuracy and uncertainty.

## Methods

### Study design and patient population

The data set comprised of imaging data of 10 patients with low-volume mHSPC who received [^177^Lu]Lu-PSMA therapy. The original prospective study was approved by the Medical Review Ethics Committee Region Arnhem-Nijmegen and was registered on clinicaltrials.gov (NCT03828838). All subjects signed an informed consent form. A comprehensive description of the patient population and clinical results has been published before [19]. In short, mHSPC patients with prostate-specific antigen (PSA) doubling time ≤ 6 months and ≤ 10 visible metastases on baseline [^68^Ga]Ga-PSMA-PET/CT, with at least one lesion ≥ 10 mm in diameter, were included. All patients underwent two cycles of [^177^Lu]Lu-PSMA therapy (cycle 1: 3.1 ± 0.1 GBq, cycle 2: 5.9 ± 0.4 GBq).

### Image acquisition

Patients received [^68^Ga]Ga-PSMA-11-PET/CT imaging approximately one week prior to radioligand therapy. Imaging was performed 60 ± 10 min post-injection (p.i.) on a Biograph mCT system (Siemens Healthineers, Erlangen, Germany) scanning cranium to trochanter major. In this study, these scans were solely used to determine lesion volumes.

After therapy, SPECT/CT and planar imaging was performed at 1, 24, 48, 72 and 168 h on either a Symbia T16 or Symbia Intevo Bold system (Siemens Healthineers, Erlangen, Germany). SPECT/CT scans were acquired at three body regions to include lesions and organs at risk: the pelvis, abdomen, and head-neck region. Acquisition and reconstruction parameters of PET/CT, SPECT/CT and planar imaging can be found in Additional file 1.

### Reference standard dosimetry

**Fig. 1:**
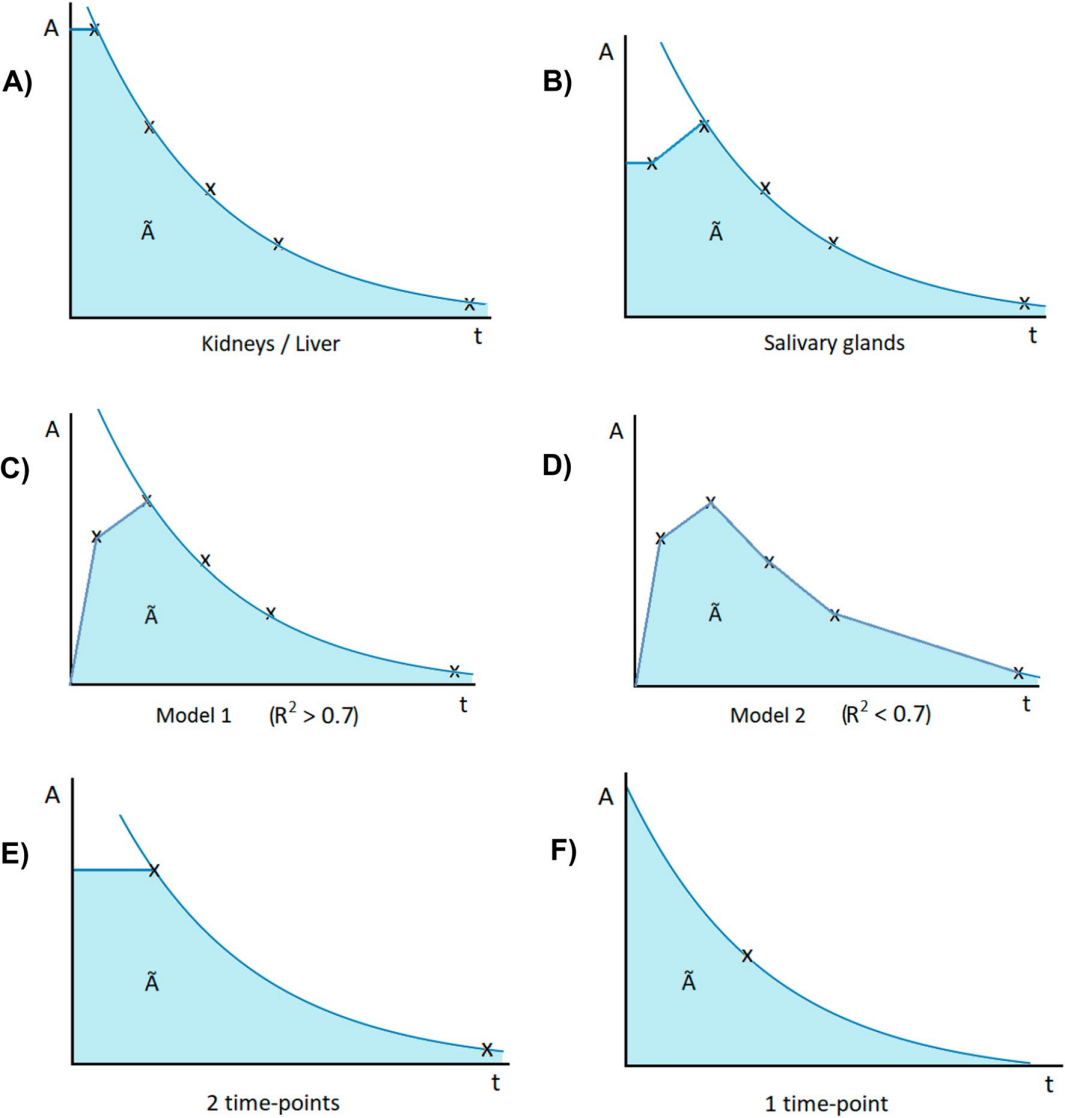
Fitting schemes for the five-time point reference standard protocol for kidneys and liver (**A**), salivary glands (**B**), lesions (**C** or **D** depending on *R*^2^), and for the simplified protocol using two time points (**E**) or one-time point (**F**)

The absorbed dose delivered to lesions, salivary glands, kidneys and liver was determined using the Medical Internal Radiation Dose (MIRD) formalism [20] based on five-time point SPECT imaging and used as reference standard absorbed dose. This methodology and the corresponding results were described before [14]. In short, organ dosimetry was performed by using reference organ weights (ICRP Publication 89 adult male human model [21]) and corresponding S-values. Counts were determined at each time point in volumes of interest (VOIs) corrected for background. Time-integrated activity in kidneys and liver was determined assuming instantaneous uptake from *t* = 0 to *t* = 1 h and mono-exponential clearance thereafter (Fig. 1A). In the salivary glands, instantaneous uptake from *t* = 0 to *t* = 1 h was assumed, followed by linear uptake to *t* = 24 h, and mono-exponential clearance thereafter (Fig. 1B). For lesions, the volume was determined on pre-therapeutic PSMA-PET/CT, either slice-by-slice on the low-dose CT, or alternatively on the PET images using an iterative thresholding method [22]. The corresponding S-value was determined using a spherical model. The time-integrated activity was determined assuming linear uptake between *t* = 0, *t* = 1 h and *t* = 24 h, and mono-exponential clearance thereafter (Fig. 1C). Alternatively, if the Pearson correlation coefficient *R*^2^ was below 0.7, a trapezoidal approach was used (Fig. 1D). For this, the tail of the curve was determined using the same rate as between time points four and five, or the physical decay rate in case this was faster. The absorbed doses per applied activity [Gy/GBq] for organs and lesions were determined for both treatment cycles separately.

### Protocol simplification

The reference absorbed dose was compared to the absorbed dose calculated from fewer time points SPECT and/or planar scans.

For one-time point SPECT dosimetry, population tissue-specific tracer kinetics had to be assumed. The mean kinetic clearance rate for that tissue was determined as the average effective decay rate of the total patient reference data set. A mono-exponential decay function was then applied to the single SPECT data point using this clearance rate (Fig. 1F).

For two-time point SPECT dosimetry, the used uptake and decay model assumed instantaneous uptake until the first considered time point, followed by the mono-exponential function connecting both time points (Fig. 1E).

### Methods of comparison

After calculating the absorbed dose based on the various compositions of time points, these compositions were compared to the reference standard absorbed dose. For this, several parameters were taken into account to evaluate whether a simplified protocol is a feasible alternative.

A Shapiro–Wilk test was used as recommended for small sample sizes. Its outcome did not show evidence for a non-normal distribution, therefore, the following parametric statistical tests were used for further analysis.

Lin’s concordance correlation coefficient *ρ*_C_ was used as a measure of agreement between the reference standard and the simplified protocol, using the following equation:

1 $$\rho_{{\text{C}}} = \frac{{2 \cdot \rho \cdot \sigma_{{{\text{ref}}}} \cdot \sigma_{{\text{s}}} }}{{\sigma_{{{\text{ref}}}}^{2} + \sigma_{{\text{s}}}^{2} + \left( {\mu_{{{\text{ref}}}} - \mu_{{\text{s}}} } \right)^{2} }}$$

with *ρ* the correlation coefficient between the reference standard absorbed dose and the absorbed dose from the simplified protocol, *σ*_ref_ and *σ*_s_ are the variance of the reference standard and the simplified protocol absorbed dose, respectively, and *µ*_ref_ and *µ*_s_ are the means of the reference standard and the simplified protocol absorbed dose, respectively. In this study, interpretation of Lin’s concordance correlation coefficient was done by setting a value of *ρ*_C_ > 0.90 as acceptable.

In addition, Bland–Altman analysis was used to compare the absorbed dose calculated from the reference standard and simplified protocols. In this analysis, the relative difference in absorbed dose between the alternative method and the reference method is compared to the mean absorbed dose of the two methods [23]. The repeatability of the simplified protocol and its prediction intervals were tested by indicating the variance in the confidence intervals.

Other parameters discussed below were first determined on a patient-specific base. Next, all parameters were determined as the mean ± standard deviation for the whole patient cohort and checked for their respective requirement as defined below.

Another important parameter to consider is the uncertainty of the absorbed dose. This was calculated according to the European Association of Nuclear Medicine (EANM) uncertainty guideline by Gear et al. [24] (for more details, see Additional file 1). Considering the clinical implications of this parameter, the maximum acceptable uncertainty was set at 25% for both lesions and organs. Furthermore, the calculated uncertainty for the reference standard absorbed dose was used as a reference.

Also, the normalized error *E*_N_ was determined for each simplified protocol, according to

2 $$E_{{\text{N}}} = \frac{{\left| {D_{{{\text{ref}}}} - D_{{\text{S}}} } \right|}}{{u\left( {D_{{{\text{ref}}}} } \right)}}$$

With *D*_ref_ and *D*_s_, the reference standard and simplified protocol absorbed dose, respectively, and *u* the uncertainty. In this equation, the absorbed dose calculated from the simplified protocol is compared to that of the reference standard, while taking into account the uncertainty of the reference dose. A perfect match is achieved for *E*_N_ = 0, so the lower the *E*_N_, the better. In combination with a minimizing constraint *u*_s_ < 0.25, *D*_s_ makes the normalized error *E*_N_ a valuable and easy interpretable test, only *E*_N_ < 1 is considered to be conforming with the reference standard.

Whether a specific simplified protocol is a feasible option is based on all parameters: the uncertainty, Lin’s concordance coefficient and the normalized error all must lie within the defined requirements.

## Results

### Reference standard dosimetry

A total of 47 lesions were defined in this study (1–7 lesions per patient). Of these, seven had an uncertainty in an absorbed dose exceeding 25% and were therefore discarded for further analysis. The median volume of the remaining 40 lesions was 0.71 ml (range 0.13–42.5 ml). Of these, 26 fulfilled the requirement of *R*^2^ > 0.7 to use a mono-exponential fit for time-integrated activity and could be used to determine a mean clearance rate for single-point lesion dosimetry (median volume: 0.68 ml, range 0.13–42.5 ml). The resulting effective half-lives for lesions (*n* = 40) and organs can be found in Table 1, as well as the median absorbed dose *D* and corresponding uncertainties *u* as calculated for the reference standard absorbed dose.

**Table 1:** Mean effective half-lives *t*_1,2,eff_ (± standard deviation (SD)), median absorbed dose D (+ range) and uncertainty *u*(*D*) for lesions and organs, following reference standard dosimetry

Structure	Mean *t*_1/2,eff_ (h)	Median *D* (Gy/GBq)	Mean *u*(*D*) (%)
Lesions	69 ± 13	2.07 (0.30–16.40)	13.6 ± 3.1
Kidneys	39 ± 5	0.52 (0.21–0.88)	15.1 ± 2.8
Liver	29 ± 8	0.08 (0.06–0.14)	21.7 ± 4.8
Salivary glands	33 ± 4	0.50 (0.15–1.28)	13.4 ± 1.8

### Simplification of the lesion dosimetry protocol

**Fig. 2:**
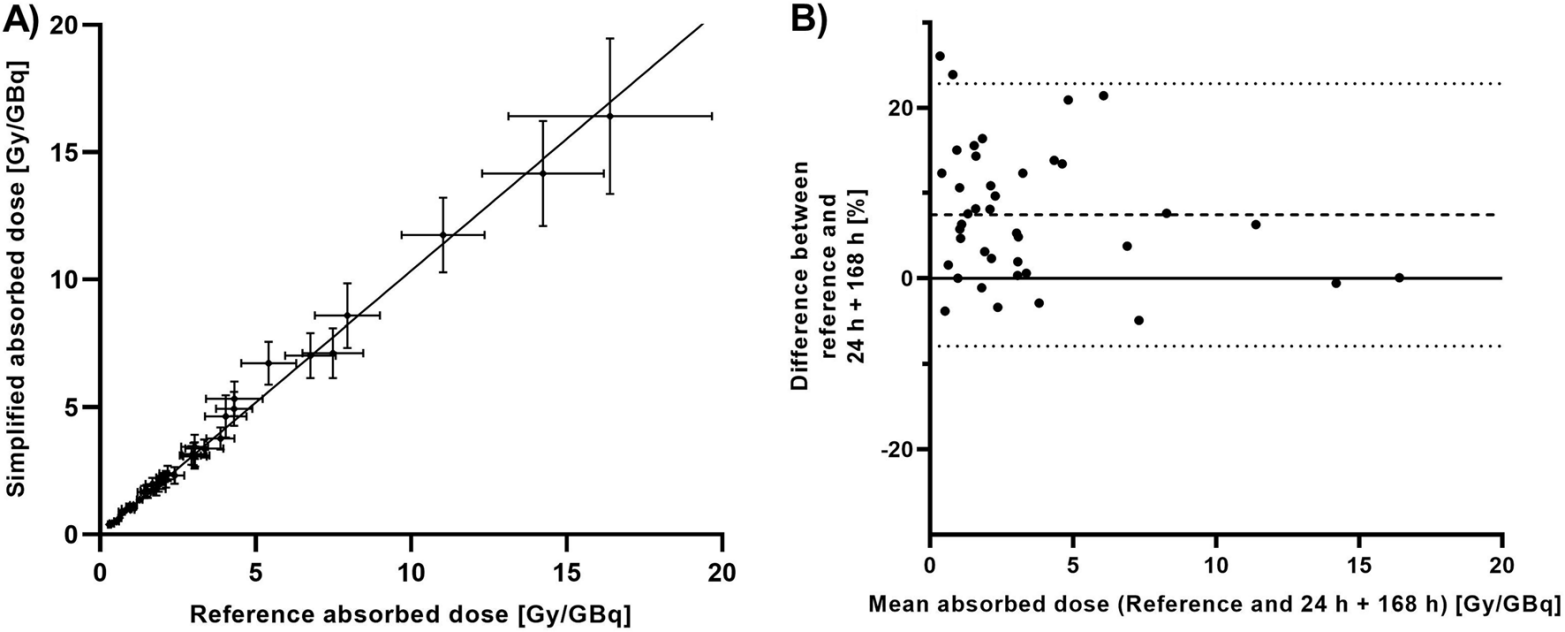
Results of lesion dosimetry based on two-time point SPECT at 24 and 168 h compared to the reference standard. **A** concordance between the absorbed dose according to the simplified protocol (*y*-axis) and the reference standard (*x*-axis). The diagonal line is the line of perfect concordance. **B** Bland–Altman plot showing the mean difference between both methods as a function of the mean absorbed dose of both methods. The upper and lower dashed lines indicate the limits of agreement

In Table 2, the comparison parameters for the dosimetry methods using one or two post-treatment SPECT scans are displayed for the lesions. Simplification protocols that meet requirements for all parameters are marked in italics. The SPECT scan at 168 h p.i. is essential for a dose estimation that is similar to the reference standard absorbed dose. Adding a second, earlier time point improves the uncertainty *u*(*D*), which is then the same as for the reference standard uncertainty (around 14%). This is visualized in Fig. 2 for the most optimal two-time point protocol using 24 h and 168 h.

**Table 2:** The uncertainty *u*(*D*), Lin’s concordance correlation coefficient *ρ*_C_ and the normalized error *E*_N_ for the simplified dosimetry protocols for the lesions

Method	*u*(*D*) (%)	*ρ*_C_	*E*_N_
1 h	**24.3 ± 1.1**	0.79	3.65 ± 1.86
24 h	**21.2 ± 1.2**	**0.96**	1.82 ± 1.32
48 h	**17.5 ± 2.3**	**0.94**	2.30 ± 1.96
72 h	**15.8 ± 1.6**	**0.94**	1.60 ± 1.79
168 h	**20.1 ± 1.3**	**0.98**	1.21 ± 0.94
1 h + 24 h	**20.3 ± 16.1**	0.66	7.24 ± 4.75
1 h + 48 h	**18.8 ± 11.6**	0.77	5.01 ± 3.69
1 h + 72 h	**16.2 ± 6.2**	0.84	3.60 ± 2.02
***1 h* + *168 h***	***13.8* ± *2.0***	***0.99***	***0.68* ± *0.61***
24 h + 48 h	31.2 ± 24.9	0.77	3.69 ± 3.12
24 h + 72 h	**18.7 ± 7.7**	**0.91**	2.27 ± 1.97
***24 h* + *168 h***	***12.9* ± *2.3***	***0.99***	***0.65* ± *0.50***
48 h + 72 h	**24.7 ± 18.1**	**0.92**	2.79 ± 1.89
***48 h* + *168 h***	***13.1* ± *3.0***	***0.99***	***0.65* ± *0.64***
***72 h* + *168 h***	***13.4* ± *2.4***	***0.99***	***0.86* ± *0.48***

Mean ± SD values are given. Parameters that meet requirements are marked in bold. Protocols where all parameters meet requirements are marked in bolditalics

### Simplification of the organ dosimetry protocol using SPECT

In Tables 3 and 4, the comparison parameters are displayed for kidneys and salivary glands, respectively. A single-time point SPECT at 24 or 48 h p.i. would be feasible to determine accurate absorbed dose for all organs of interest. In Additional file 1: Tables S1–S3, a complete overview including liver results and other time point combinations can be found, showing that some other two-time point protocols could be used as well.

**Table 3:** The uncertainty *u*(*D*), Lin’s concordance correlation coefficient *ρ*_C_ and the normalized error *E*_N_ for the simplified dosimetry protocols for the kidneys

Method	*u*(*D*) (%)	*ρ*_C_	*E*_N_
1 h	**17.3 ± 0.1**	0.48	2.23 ± 1.43
***24 h***	***14.3* ± *0.3***	***0.94***	***0.73* ± *0.87***
**48 h**	**12.3 ± 0.1**	0.80	**0.99 ± 0.80**
72 h	**12.7 ± 0.1**	0.85	1.06 ± 0.25
168 h	27.5 ± 3.9	0.75	1.63 ± 1.42
1 h + 168 h	**11.6 ± 0.1**	0.65	2.03 ± 1.92
***24 h* + *168 h***	***11.4* ± *0.1***	***0.97***	***0.44* ± *0.51***
48 h + 168 h	**11.3 ± 0.1**	0.73	1.58 ± 0.22
72 h + 168 h	**11.2 ± 0.1**	0.43	2.64 ± 0.32

Mean ± SD values are given. Parameters that meet requirements are marked in bold. Protocols where all parameters meet requirements are marked in bolditalics

**Table 4:** The uncertainty *u*(*D*), Lin’s concordance correlation coefficient *ρ*_C_ and the normalized error *E*_N_ for the simplified dosimetry protocols for the salivary glands

Method	*u*(*D*) (%)	*ρ*_C_	*E*_N_
***1 h***	***17.1* ± *0.2***	***0.95***	***0.87* ± *0.54***
24 h	**13.8 ± 0.3**	**0.91**	1.50 ± 0.96
***48 h***	***12.2* ± *0.0***	***0.99***	***0.56* ± *0.32***
***72 h***	***13.8* ± *0.3***	***0.95***	***0.95* ± *0.52***
168 h	33.4 ± 5.3	0.86	1.55 ± 1.09
***1 h* + *168 h***	***11.6* ± *0.1***	***0.98***	***0.66* ± *0.34***
***24 h* + *168 h***	***11.4* ± *0.1***	***0.98***	***0.70* ± *0.59***
48 h + 168 h	**11.2 ± 0.0**	0.82	1.95 ± 0.32
72 h + 168 h	**11.2 ± 0.0**	0.45	3.67 ± 0.40

Mean ± SD values are given. Parameters that meet requirements are marked in bold. Protocols where all parameters meet requirements are marked in bolditalics

**Fig. 3:**
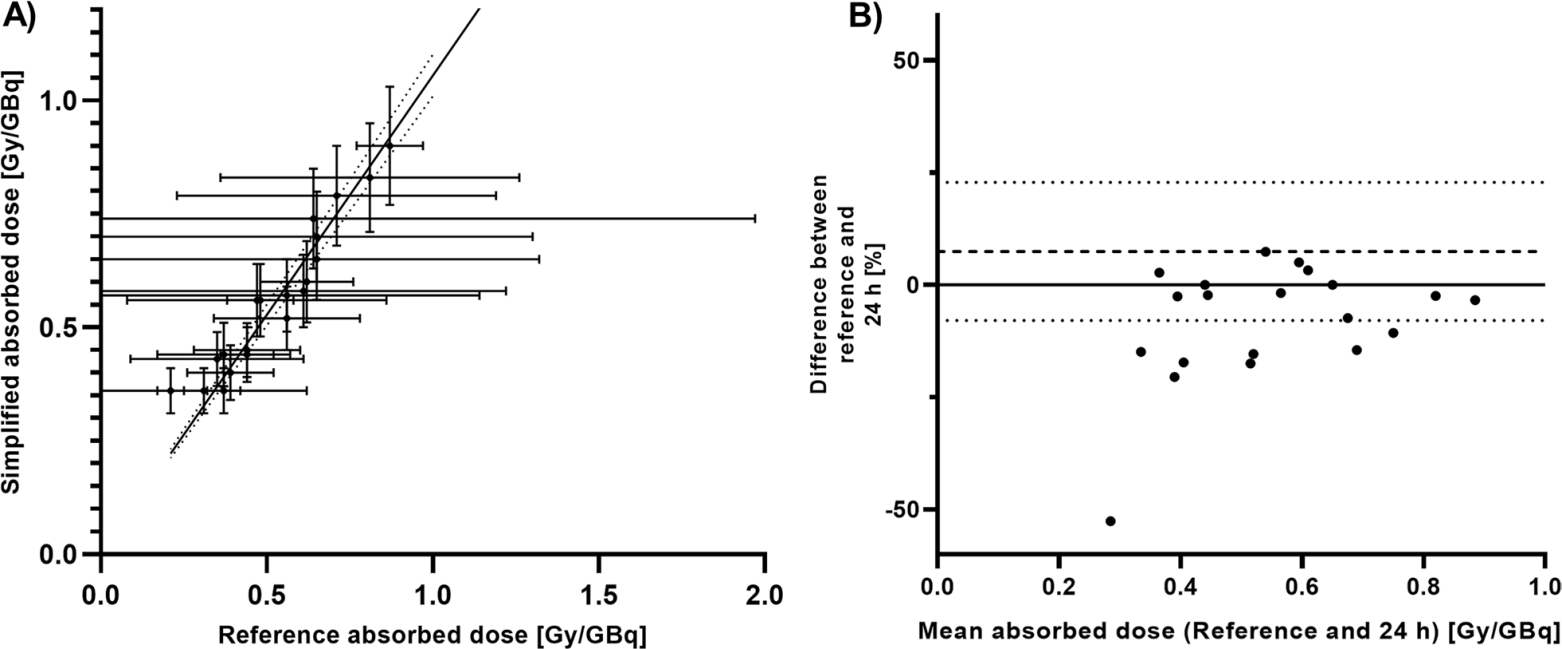
Results of organ dosimetry based on a single SPECT at 24 h compared to the reference standard for kidneys. **A** concordance between the absorbed dose according to the new method (*y*-axis) and the reference standard (*x*-axis). The diagonal line is the line of perfect concordance. **B** Bland–Altman plot showing the mean difference between both methods as a function of the mean absorbed dose of both methods. The upper and lower dashed lines indicate the limits of agreement

**Fig. 4:**
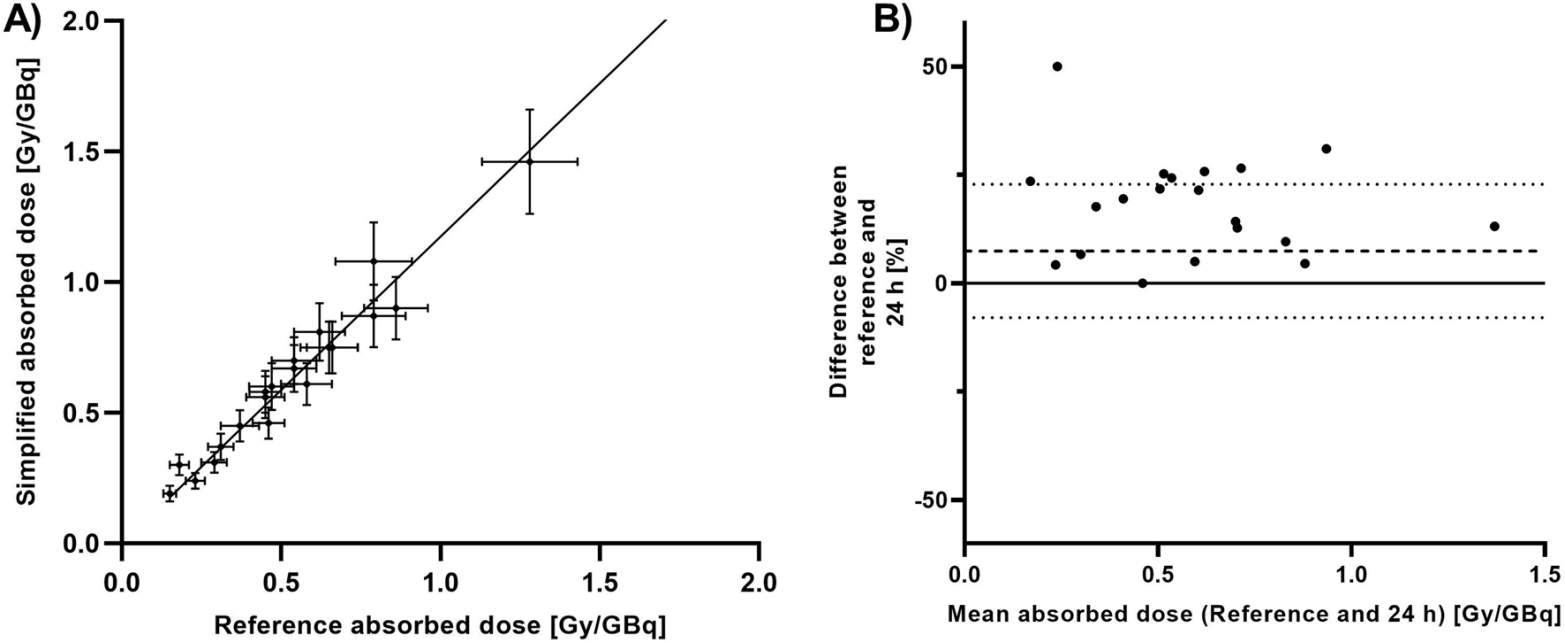
Results of organ dosimetry based on a single SPECT at 24 h compared to the reference standard for salivary glands. **A** concordance between the absorbed dose according to the new method (*y*-axis) and the reference standard (*x*-axis). The diagonal line is the line of perfect concordance. **B** Bland–Altman plot showing the mean difference between both methods as a function of the mean absorbed dose of both methods. The upper and lower dashed lines indicates the limits of agreement

A visualization of these results for kidneys and salivary glands of the 24 h protocol can be found in Figs. 3 and 4. Absorbed dose calculation for salivary glands could be improved by including a second scan at 168 h, or by using a single-time point at 48 h p.i. (Additional file 1: Fig. S1).

### Simplification of the organ dosimetry protocol using whole-body planar scans

Planar scan dosimetry was only considered for the organs, since (small) lesions were mostly not visible on the planar scans and time-integrated activity could therefore not be determined.

For simplification of the imaging protocol for organs using planar scans, first, the absorbed dose determined from the five-time point planar protocol was compared to the reference absorbed dose (Table 5). For the kidneys and salivary glands, the absorbed dose was consistently overestimated, while for the liver, it was underestimated. A correction factor was determined for each organ by fitting a linear function to the scatter plots (Additional file 1: Fig. S2) crossing the origin. However even after application of the correction factor, the planar protocol did not fulfill all requirements for any of the organs. This suggests a poor estimation of tracer effective half-life based on planar scans. This could not be attributed to poor organ volume estimation, since in this study, we used reference volumes based on the ICRP89 male adult model, and thus, these volumes were equal for SPECT and planar dosimetry. Therefore, we concluded that a dosimetry protocol using only planar scans does not yield accurate dose estimation for organs at risk, and protocols with fewer planar scans were not further investigated.

**Table 5:** The uncertainty *u*(*D*), Lin’s concordance correlation coefficient *ρ*_C_ and the normalized error *E*_N_ for the simplified planar dosimetry protocols for the organs

Method	Organ	Correction factor	*u*(*D*) (%)	*ρ*_C_	*E*_N_
Original 5 time point planar	Kidneys	–	**18.2 ± 6.4**	0.17	2.83 ± 1.30
	Liver	–	52.1 ± 15.7	0.56	**0.64 ± 0.46**
	Salivary glands	–	**12.9 ± 1.6**	0.14	4.21 ± 1.35
Planar with correction factor	Kidneys	0.47	**18.2 ± 6.4**	0.63	1.01 ± 0.97
	Liver	1.27	52.1 ± 15.7	0.65	**0.44 ± 0.28**
	Salivary glands	0.36	**12.9 ± 1.6**	0.40	1.36 ± 1.43

Mean ± SD values are given. Parameters that meet requirements are marked in bold

## Discussion

In this study, we aimed to improve the clinical imaging protocol for [^177^Lu]Lu-PSMA dosimetry, by balancing between accuracy and uncertainty of absorbed dose assessment, and practical considerations such as patient burden and use of hospital resources. A simplified dosimetry protocol could aid in the implementation of dosimetry into the clinical routine, thereby providing a tool to personalize [^177^Lu]Lu-PSMA therapy. This will improve patient selection and treatment response, reduce toxicity and keep costs under control.

In this study, we have demonstrated that the dosimetry protocol can be simplified with a slight loss of reliability. This is also in line with what was found in simplification studies concerning [^177^Lu]Lu-DOTATATE treatment in neuroendocrine tumors [17, 18]. Different imaging protocols were used to determine the absorbed dose and compared to the absorbed dose from an elaborate five-time point SPECT protocol (the reference standard). For reliable lesion dosimetry, a late time point SPECT scan such as 168 h p.i. is crucial, which can be further improved in terms of uncertainty by adding a second SPECT scan at 24 or 48 h. For organs, single-time point dosimetry using an early time point (24 h or 48 h) proved to be feasible, which could be improved by adding a second late time point SPECT scan. While using planar imaging for dosimetry would mean a significant reduction in scan time, we showed that no reliable dosimetry could be performed based on planar imaging only. This is an important finding, since many dosimetry studies performed so far in [^177^Lu]Lu-PSMA therapy have used planar scan dosimetry [11, 13, 16]. Since 3D SPECT dosimetry is known to be superior to planar scan dosimetry, and only one or two time points are sufficient for reliable dosimetry, it is strongly suggested that future dosimetry studies use SPECT imaging as the method of choice. If feasible, we suggest to limit the number of bed positions to a maximum of two per time point (for example by choosing a bed position that includes both the kidneys and (some) major lesions of interest). In this regard, a combined planar and SPECT/CT approach does not seem to be of interest, since one-time point SPECT already yields a reliable dosimetry protocol for this therapy.

This study was based on only 10 patients and 20 therapy cycles, therefore, our findings should be verified with further research. However, we still concluded that this data set is suitable to simplify the protocol, as variations in organ kinetics between patients were minimal [14]. Our reference dataset comprised of five-time point SPECT imaging, so the tracer kinetics for various tissues could be followed in detail. However, the information on early time points was limited to the scan at 1 h post-injection, therefore, the assumption on early uptake kinetics should be verified with additional data. Also, it should be stressed that the goal of this research was to find a suitable simplified scanning scheme, so the focus was on comparing the reference standard to the simplified protocol and not on finding the optimal dosimetry protocol for the reference standard. Still, our reference standard protocol included three-bed positions SPECT/CT at five imaging time points. As this is a very elaborate imaging scheme for dosimetry purposes, we believe this protocol is suitable to serve as the reference standard for the simplification. Also, bone marrow dosimetry was not included in this simplification study. While bone marrow toxicity is an important concern in [^177^Lu]Lu-PSMA therapy, setting up a simplified protocol for bone marrow dosimetry requires further investigation, including verification of the possibility to use imaging for bone marrow dosimetry in this specific treatment. This is part of a current study conducted by the authors. Several models are available to determine the absorbed dose to the bone marrow. The blood-based model [25, 26] is quite simple to implement, making it the most applied method. This model, however, is only valid for patients without specific uptake in the marrow space, either in bone metastases or through in vivo dechelation when an endogenous ligand as transferrin displaces the DOTA chelator [27, 28]. More complex models taking the actual bone marrow reserve and potential displacement of red marrow around bone metastases are available but not commonly applied [29]. The current method for bone marrow dosimetry is only applicable to account for the absorbed dose by blood flow through the marrow space, without specific uptake.

We evaluated [^177^Lu]Lu-PSMA-617 dosimetry simplification in mHSPC patients. Since we previously showed that for this patient cohort, tracer uptake kinetics in organs are comparable to those in metastasized castrate-resistant prostate cancer (mCRPC) patients [14], we postulate that the results of this study could be translated to dosimetry in mCRPC patients as well. This is also supported by recent findings of comparable studies in this patient population [30–33]. For lesion dosimetry, a two-time point protocol is proposed; so, no general kinetics apart from a single compartment distribution are assumed. While a future study needs to verify the optimal time points for lesion dosimetry in mCRPC patients, the present results are in line with a study by Jackson and colleagues, showing tumor dose estimates based on single-time point SPECT dosimetry were most accurate using delayed scanning at time points beyond 72 h p.i. [34].

In this study, we evaluated all different combinations of time points, so the user can decide which imaging protocol is most suitable for their clinical setting. But, based on the requirements set in this study, we suggest a dosimetry protocol using three-bed position SPECT/CT at 24 h including both organs and lesions, and an additional one-bed position SPECT/CT at 168 h for the lesions. However, depending on the specific question, clinical grounds or practical considerations, imaging at other time points, could also provide an alternative without significant loss of accuracy. The proposed methodology for protocol simplification can be applied universally, both to evaluate different patient cohorts in prostate cancer, as well as the use of different PSMA tracers (for example PSMA-11, PSMA-1007 and PSMA-I&T instead of PSMA-617), and even for different radionuclide therapies.

Given the results of this work, we propose to apply this protocol to pre-therapeutic PET-based dosimetry to improve patient selection for [^177^Lu]Lu-PSMA therapy. To date, many different PSMA ligands labeled with long-lived PET isotopes have been investigated for prostate cancer imaging, such as [^89^Zr]Zr-PSMA [35, 36]. By including an additional scan to the standard clinical protocol, these diagnostic scans can be used for dosimetry with acceptable costs and low patient burden. In this way, patient selection can be considerably improved by only including patients with expected high tumor absorbed doses and acceptable absorbed doses to organs at risk. Considering that [^177^Lu]Lu-PSMA therapy is moving to earlier disease stages and is likely to exceed 4–6 treatment cycles in patients, proper patient selection and a more individualized dosing plan will become crucial in the near future. This more personalized approach will optimize therapeutic effect, minimize toxicity, keep costs under control and improve our understanding of radioligand therapy.

## Conclusion

Dosimetry is needed to improve patient selection for [^177^Lu]Lu-PSMA therapy. In the present study, we simplified the [^177^Lu]Lu-PSMA dosimetry protocol. Our results indicate that dosimetry can be reliably performed using a limited number of scans. By reducing the number of scans, there will be less burden to patients and demand for hospital resources which is needed for a broader adoption of dosimetry into clinical practice.

## Supplementary Information

**Additional file 1. S1**: Acquisition and reconstruction parameters of the imaging protocols. **S2**: Uncertainty analysis. **S3**: The uncertainty u(D), Lin’s concordance correlation coefficient ρC and the normalized error EN for the simplified dosimetry protocols for the kidneys. **S4**: The uncertainty u(D), Lin’s concordance correlation coefficient ρC and the normalized error EN for the simplified dosimetry protocols for the liver. **S5**: The uncertainty u(D), Lin’s concordance correlation coefficient ρC and the normalized error EN for the simplified dosimetry protocols for the salivary glands. **S6**: Bland–Altman plots for salivary glands. **S7**: Concordance between the absorbed dose based on five post-treatment planar scans and the reference standard for kidneys, liver and salivary glands, without and with correction factor.

## Data Availability

The datasets generated during and/or analyzed during the current study are available from the corresponding author on reasonable request.

## Abbreviations

CT: Computed tomography
EANM: European Association of Nuclear Medicine
MIRD: Medical Internal Radiation Dose
mCRPC: Metastatic castrate-resistant prostate cancer
mHSPC: Metastatic hormone-sensitive prostate cancer
mPC: Metastatic prostate cancer
p.i.: Post-injection
PET: Positron emission tomography
PSA: Prostate specific membrane antigen
PSMA: Prostate specific membrane antigen
SPECT: Single photon emission computed tomography
VOI: Volume of interest
