# Supplementary material for: Optimization of the radiation dosimetry protocol in Lutetium-177-PSMA therapy: toward clinical implementation
Source: EJNMMI Res. 2023 Jan 24;13:6. doi: 10.1186/s13550-023-00952-z (PMC9873880; doi:10.1186/s13550-023-00952-z)
Supplement: Supplementary file 1 — Additional file 1. S1: Acquisition and reconstruction parameters of the imaging protocols. S2: Uncertainty analysis. S3: The uncertainty u(D), Lin’s concordance correlation coefficient ρC and the normalized error EN for the simplified dosimetry protocols for the kidneys. S4: The uncertainty u(D), Lin’s concordance correlation coefficient ρC and the normalized error EN for the simplified dosimetry protocols for the liver. S5: The uncertainty u(D), Lin’s concordance correlation coefficient ρC and the normalized error EN for the simplified dosimetry protocols for the salivary glands. S6: Bland–Altman plots for salivary glands. S7: Concordance between the absorbed dose based on five post-treatment planar scans and the reference standard for kidneys, liver and salivary glands, without and with correction factor. [file 13550_2023_952_MOESM1_ESM.docx]

Supplementary materials

**Title: Optimization of the Radiation Dosimetry Protocol in Lutetium-177-PSMA Therapy: towards Clinical Implementation**

**Authors:** Steffie M.B. Peters^1,*^, Maaike C.T. Mink^1,2,*^, Bastiaan M. Privé^1^, Maarten de Bakker^1^, Frank de Lange^1^, Constantijn H.J. Muselaers^3^, Niven Mehra^4^, J. Alfred Witjes^3^, Martin Gotthardt^1^, James Nagarajah^1,¤^, Mark W. Konijnenberg^1,5, ¤^

^1^: Department of Medical Imaging, Radboud university medical center, Nijmegen, The Netherlands

^2^: Department of Physics and Astronomy, Radboud University, Nijmegen, The Netherlands

^3^: Department of Urology, Radboud university medical center, Nijmegen, The Netherlands

^4^: Department of Medical Oncology, Radboud university medical center, Nijmegen, The Netherlands

^5^: Department of Radiology and Nuclear Medicine, Erasmus Medical Center, Rotterdam, The Netherlands

*, ^¤^: these authors contributed equally to this work

**Corresponding and first author:**

Steffie M.B. Peters (ORCID: 0000-0002-0752-7134)

Radboud university medical center

Department of Medical Imaging

P.O. Box 9101

6500 HB Nijmegen, The Netherlands

T: +31 (6) 11621752

E: [steffie.peters@radboudumc.nl](mailto:steffie.peters@radboudumc.nl)

**Supplementary Materials 1: Acquisition and reconstruction parameters of the imaging protocols**

**[^68^Ga]Ga-PSMA -11-PET/CT**

Patients received [^68^Ga]Ga-PSMA -11-PET/CT prior to radioligand therapy. Imaging was performed 60 ± 10 minutes post-injection on a Biograph mCT system (Siemens Healthineers, Erlangen, Germany) scanning cranium to trochanter major. For the pelvis region, data were acquired using 4 minutes per bed position, whereas 3 minutes per bed position were applied for the other regions. PET data were reconstructed using ordinary Poisson ordered-subset expectation maximization with time-of-flight modelling (OP-OSEM-TOF) with 2 iterations and 21 subsets, matrix size of 400 (resulting in a cuboid-shape voxel size of approximately 8.0 (2.0 x 2.0 x 2.0) mm^3^) and a smoothing Gaussian filter of 3 mm. The estimated reconstructed PET spatial resolution expressed as full width at half maximum (FWHM) was 6.3 mm. A low dose CT was performed (average dose length product (DLP) of 291 mGy·cm) and the CT data were reconstructed using 3.0 mm slice thickness and kernel B19f; the reconstructed transverse CT images had a voxel size of 1.0 x 1.0 x 3.0 mm^3^. For all PET images, standard corrections for CT-based attenuation, scatter, decay, and dead-time were performed.

**[^177^Lu]Lu-PSMA SPECT/CT**

SPECT/CT and planar imaging was performed at 1, 24, 48, 72 and 168 hours after administration of [^177^Lu]Lu-PSMA on either a Symbia T16 or Symbia Intevo Bold system (Siemens Healthineers, Erlangen, Germany). SPECT/CT scans were acquired at three body regions: the pelvis, abdomen, and head-neck region (64 projections per detector, time per projection of 14 s, a 20% photon energy window at 208 keV, and dual-energy window for Compton scattering. SPECT data were reconstructed using OSEM reconstruction (Flash 3D with collimator detector response) using 4 iterations and 8 subsets, matrix size of 128 (resulting in a cuboid-shape voxel size of 4.8 mm^3^) and a smoothing Gaussian filter of 8.4 mm. The estimated reconstructed SPECT spatial resolution was 15 mm (FWHM). A low dose CT was performed (average DLP of 130 mGy·cm ) and the data were reconstructed using B31s kernel and 3.0 mm slice thickness resulting in a voxel size of 1.0 x 1.0 x 3.0 mm^3^. Of note, the SPECT image reconstruction approach takes into account corrections for scatter, CT-based attenuation, and dead-time.

Whole body planar imaging was performed using a 10 cm/min scan rate and a standard scan length of 200 cm. At every time point, a standard source was placed between the patient’s legs to determine the calibration factor for each patient.

**Supplementary Materials 2: Uncertainty analysis**

**Data collection:**

- 1. Quantitative SPECT at 5 time points, 3 bed positions
  2. Quantitative PET at 1 time point

**Uncertainty in SPECT cumulated activity**

1. Error in SPECT camera specific calibration factor for ^177^Lu: 5% [Peters, 2020]

CF = 10.6 ± 0.5 cps/MBq

1. Drawing of VOIs in SPECT data over organs and tumor lesions to determine counts:
2. Large spherical VOI over lesions with background correction; counts error: 10%
3. CT based VOI over organs; counts error 5%
4. Determine Poisson-based uncertainty in counts:

$u\left( counts \right)= \frac{1}{\sqrt{counts}}$

5. Fit to Time-Activity Curve on SPECT
6. Mono-exponential fit $A\left( t \right)=A_{0}e^{-\lambda_{eff}t}$ between activity on each subsequent SPECT time point, considered an approximation of trapezoid integration
7. Integration over time of the TAC, to determine the time-integrated activity Ã on SPECT (reference absorbed dose); error:

$$\left[ \frac{\delta\tilde{A}}{\tilde{A}} \right]=\sqrt{\left[ \frac{{\delta A}_{0}}{A_{0}} \right]^{2}+\left[ \frac{{\delta\lambda}_{eff}}{\lambda_{eff}} \right]^{2}+2\frac{u\left( A_{0},\lambda_{eff} \right)}{A_{0} \lambda_{eff}}}$$

8. Trapezoid integration method; error:

$$\left[ \frac{\delta\tilde{A}}{\tilde{A}} \right]^{2}=\sum_{i} \left[ \frac{\delta A(t_{i})}{A(t_{i})} \right]^{2}$$

**General uncertainty**

1. Error in SPECT camera specific calibration factor for ^177^Lu: 5% [Peters, 2020]

CF = 10.6 ± 0.5 cps/MBq

1. Determination of lesion and organ volume:
2. Organ volume based on ICRP Publication 89 adult male human model;

Estimation of variance of actual patient’s organ volume with respect to ICRP Publication 89 adult male human model: 10%

1. Lesion volume based on CT;

Voxelization and resolution error volume with lesion diameter d and voxel size a: $\left[ \frac{\delta V}{V} \right]^{2}=9 \left[ \frac{\delta d}{d} \right]^{2}=1.5\left( \frac{a}{d} \right)^{2}$ [Gear, 2018]

1. Lesion volume based on PET [Jentzen, 2015]: uncertainty 10%
2. Determine volume specific lesion S-factor with power-function on S-values spheres $S=81.7V^{-0.988}$; error:

$$\left[ \frac{\delta S}{S} \right]^{2}=\left( 0.988\frac{\delta V}{V} \right)^{2}$$

3. Absorbed dose calculation with MIRD equation $D=\tilde{A}\times S$; error:

$$\left[ \frac{\delta D}{D} \right]^{2}={\left[ \frac{\delta\tilde{A}}{\tilde{A}} \right]^{2}+\left[ \frac{\delta S}{S} \right]}^{2}+2\frac{u\left( \tilde{A},S \right)}{\tilde{A} S}$$

where the last term is zero since the VOIs used for Ã and volume (S) are independent as large VOIs are used for quantification and the volumes are based on independent PET, phantom or CT data.

**Supplementary Materials** 3: The uncertainty u(D), Lin’s concordance correlation coefficient **ρ_C_** and the normalized error E_N_ for the simplified dosimetry protocols for the kidneys. Mean ± SD values are given. Parameters that meet requirements are marked in green. Protocols where all three parameters meet requirements are marked in grey.

| **Kidneys** | | | |
| --- | --- | --- | --- |
| **Method** | **u(D) (%)** | **ρ_C_** | **E_N_** |
| 1 h | **17.3 ± 0.1** | 0.48 | 1.17 ± 0.68 |
| **24 h** | **14.3 ± 0.3** | **0.94** | **0.45 ± 0.50** |
| **48 h** | **12.3 ± 0.1** | 0.80 | **0.78 ± 0.51** |
| **72 h** | **12.7 ± 0.1** | 0.85 | **0.88 ± 0.26** |
| 168 h | 27.5 ± 3.9 | 0.75 | **0.57 ± 0.36** |
| 1 h + 24 h | **18.0 ± 7.6** | 0.69 | **0.77 ± 0.41** |
| 1 h + 48 h | **12.4 ± 1.9** | 0.61 | **0.66 ± 0.58** |
| **1 h + 72 h** | **11.7 ± 0.1** | **0.95** | **0.32 ± 0.26** |
| 1 h + 168 h | **11.6 ± 0.1** | 0.65 | 1.18 ± 0.67 |
| 24 h + 48 h | **12.3 ± 0.7** | 0.30 | 1.38 ± 1.02 |
| **24 h + 72 h** | **11.5 ± 0.1** | 0.86 | **0.82 ± 0.23** |
| **24 h + 168 h** | **11.4 ± 0.1** | **0.97** | **0.32 ± 0.38** |
| 48 h + 72 h | **12.9 ± 1.4** | 0.56 | 1.87 ± 0.40 |
| 48 h + 168 h | **11.3 ± 0.1** | 0.73 | 1.39 ± 0.25 |
| 72 h + 168 h | **11.2 ± 0.1** | 0.43 | 2.44 ± 0.35 |

**Supplementary Materials** 4: The uncertainty u(D), Lin’s concordance correlation coefficient **ρ_C_** and the normalized error E_N_ for the simplified dosimetry protocols for the liver. Mean ± SD values are given. Parameters that meet requirements are marked in green. Protocols where all three parameters meet requirements are marked in grey.

| **Liver** | | | |
| --- | --- | --- | --- |
| **Method** | **u(D) (%)** | **ρ_C_** | **E_N_** |
| 1 h | 29.9 ± 0.4 | 0.24 | 1.02 ± 0.30 |
| **24 h** | **17.8 ± 1.3** | 0.81 | **0.46 ± 0.32** |
| **48 h** | **13.0 ± 0.5** | 0.77 | **0.74 ± 0.32** |
| 72 h | **24.3 ± 1.2** | 0.64 | **0.80 ± 0.31** |
| 168 h | 85.1 ± 17.5 | 0.03 | **0.54 ± 0.14** |
| **1 h + 24 h** | **12.8 ± 0.7** | **0.91** | **0.36 ± 0.26** |
| **1 h + 48 h** | **11.6 ± 0.1** | 0.89 | **0.24 ± 0.29** |
| **1 h + 72 h** | **11.6 ± 0.0** | 0.87 | **0.41 ± 0.33** |
| 1 h + 168 h | **11.7 ± 0.1** | 0.18 | 2.20 ± 0.59 |
| 24 h + 48 h | **11.8 ± 1.3** | 0.67 | **0.91 ± 0.29** |
| **24 h + 72 h** | **11.3 ± 0.0** | 0.79 | **0.62 ± 0.24** |
| **24 h + 168 h** | **11.4 ± 0.1** | 0.70 | **0.62 ± 0.49** |
| 48 h + 72 h | **12.1 ± 1.9** | 0.27 | 2.00 ± 0.48 |
| 48 h + 168 h | **11.4 ± 0.6** | 0.44 | 1.37 ± 0.40 |
| 72 h + 168 h | **11.3 ± 0.1** | 0.13 | 2.37 ± 0.52 |

**Supplementary Materials** 5: The uncertainty u(D), Lin’s concordance correlation coefficient **ρ_C_** and the normalized error E_N_ for the simplified dosimetry protocols for the salivary glands. Mean ± SD values are given. Parameters that meet requirements are marked in green. Protocols where all three parameters meet requirements are marked in grey.

| **Salivary Glands** | | | |
| --- | --- | --- | --- |
| **Method** | **u(D) (%)** | **ρ_C_** | **E_N_** |
| **1 h** | **17.5 ± 0.2** | **0.95** | **0.52 ± 0.36** |
| **24 h** | **13.8 ± 0.3** | **0.91** | **0.90 ± 0.53** |
| **48 h** | **12.2 ± 0.0** | **0.99** | **0.40 ± 0.26** |
| **72 h** | **13.8 ± 0.3** | **0.95** | **0.72 ± 0.44** |
| 168 h | 33.4 ± 5.3 | 0.86 | **0.48 ± 0.27** |
| 1 h + 24 h | 26.0 ± 13.4 | 0.25 | 1.96 ± 1.93 |
| **1 h + 48 h** | **12.3 ± 0.6** | **0.98** | **0.43 ± 0.23** |
| **1 h + 72 h** | **11.6 ± 0.1** | **0.98** | **0.48 ± 0.15** |
| **1 h + 168 h** | **11.6 ± 0.1** | **0.98** | **0.49 ± 0.28** |
| **24 h + 48 h** | **12.2 ± 0.8** | **0.98** | **0.51 ± 0.41** |
| **24 h + 72 h** | **11.4 ± 0.1** | **0.98** | **0.52 ± 0.28** |
| **24 h + 168 h** | **11.4 ± 0.1** | **0.98** | **0.52 ± 0.40** |
| 48 h + 72 h | **11.8 ± 0.5** | 0.72 | 2.22 ± 0.30 |
| 48 h + 168 h | **11.2 ± 0.0** | 0.82 | 1.64 ± 0.29 |
| 72 h + 168 h | **11.2 ± 0.0** | 0.45 | 3.35 ± 0.40 |


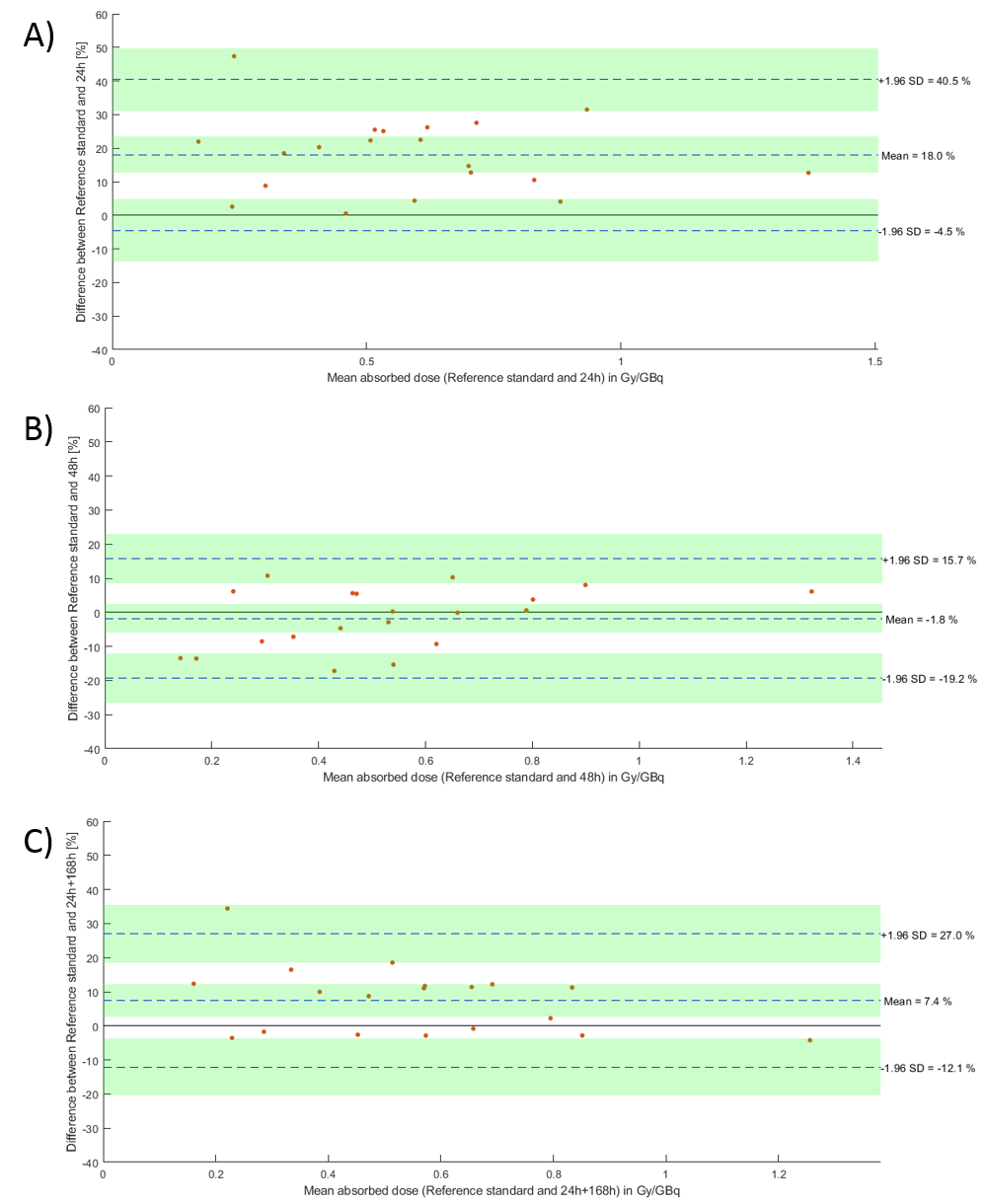


**Supplementary Materials** 6: Bland-Altman plots for salivary glands. A) Absorbed dose calculated from a single SPECT at 24 h p.i.; B) Absorbed dose calculated from a single SPECT at 48 h p.i.; C) Absorbed dose calculated from two-time point SPECT at 24 h and 168 h. As can be appreciated from these plots, absorbed dose calculation based on a 48 h SPECT scan or based on 24 h + 168 h SPECT yields a more reliable dose estimation then based on a 24 h SPECT scan only.


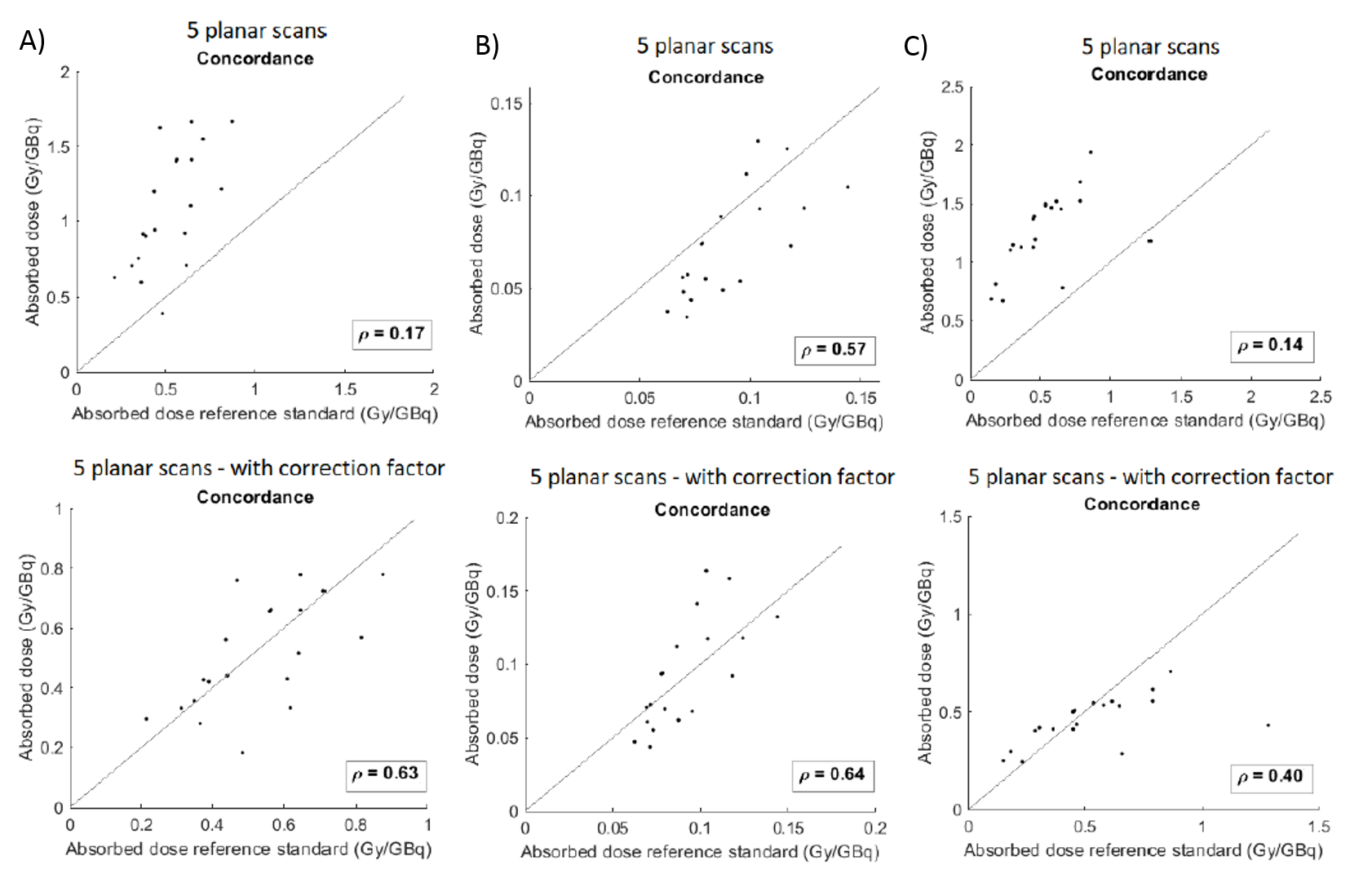


**Supplementary Materials** 7: Concordance between the absorbed dose based on five post-treatment planar scans (y-axis) and the reference standard (x-axis) for kidneys (A), liver (B) and salivary glands (C), without (top) and with (bottom) correction factor. The diagonal line is the line of perfect concordance.
